# Pelvic Cellulitis

**Published:** 1882-11

**Authors:** Jno. G. Earnest

**Affiliations:** Atlanta, Ga.


ATLANTA
### MEDICAL REGISTER.

VOL. II.] NOVEMBER, 1882. [No. 2

### Original.

### PELVIC CELLULITIS.

BY JNO. G. EARNEST, M.D., ATLANTA, GA.

*Read Before the Atlanta Medical Society.*

Several terms have been in use, from time to time, to designate inflammation of the cellular tissue of the pelvis. Perimetritis, periuterine phlegmon, pelvic abscess, pelvic cellutis and periuterine cellulitis have in turn had their day and special adherents. At present the term pelvic cellulitis, introduced by Sir James Simpson, is very generally used although Thomas insists upon periuterine cellulitis as a term of more definite meaning. As, however, there seems to be little ground for the fear of confusion with psoas abscess and other affections belonging to general surgery, we will adhere to pelvic cellulitis as the term best describing the condition.

There are cases reported by Archigenes in the second century and during the sixth and seventh centuries by Paul of Ægina and others although their knowledge of its pathology was doubtless very meagre. From this time on we find no further allusion to it until Richard Wiseman, of England, in 1679 describes some cases under the head of "distempers of the womb in child bed." Then follows a gap of two centuries in the literature of the subject when in 1841 Bourdon, of France, brought the subject prominently before the profession and was soon followed by others in France, England and Ireland.

An attack of pelvic cellulitis is usually ushered in by a rigor more or less well marked, followed by a rise in temperature and a rapid, but not generally very full pulse. There will, however, sometimes be congestion, with great tenderness and a well marked boggy feeling of the tissues involved for a day or two before the rise in temperature and pulse rate comes. This is especially true of cases that have been kept well under the influence of opiates. The patient complains of pain in the back and around the hips. There is well marked tenderness over the lower part of the abdomen and especially over one or both ovaries. The pain is increased by moving the lower limbs or assuming the erect posture. This is a fair outline of an ordinary typical case, but in actual practice we find many variations. For instance I was called to see a married lady about thirty years old who was still up looking after her affairs. She had walked half a mile the day I was called in. Four weeks since she was so unwell for a few days that she was compelled to lie down a portion of the time; was troubled with alternate rigors and flushes of heat; had pain in her limbs and back. Since that time she has been annoyed with constant pain in her hips and back accompanied with occasional flushes of heat although she has managed to keep up. Her bowels were constipated and gave much pain when they moved. An examination per vaginam found the uterus titled backward and firmly fixed. There was a smooth hard mass occupying a portion of the roof of the vagina on the left side and extending well in behind the uterus. She was put to bed. Had morning and evening a hot water injection lasting half an hour. The abdomen was painted with tincture of iodine. She had a suppository of opium as often as necessary to relieve pain. Her diet was simple and nutritious, avoiding bulky food. She made a good recovery—the tumor disappearing after several weeks.

As to the origin of pelvic cellulitis Mathews Duncan says it is never idiopathic, but is invariably secondary either to mechanical injury or the extension of inflammation of some of the pelvic viscera or to the irritation of the noxious discharges through or from the tubes or ovaries. Playfair remarks that it is pretty generally admitted by all modern writers that both pelvic peritonitis and pelvic cellulitis are produced by extension of inflammation from either the uterus, fallopian tubes or ovaries. Such a statement seems to be entirely unphilosophical. Even the weight of such justly great names has failed to carry conviction to some of us on this point. We certainly have idiopathic inflammation of the cellular tissue of other parts of the body as shown in the old fashioned phlegmon and other affections. Why not of the pelvic cellular tissue also? There is nothing about it that releases it from the laws that govern inflammation in other regions. Emmet gives the history of three hundred and three cases treated in his private hospital. He says "of the total number treated 157 or 51.81 per cent. had no uterine or ovarian disease that could be detected." Of the 157 cases he attributed nineteen to exposure to cold, two to excessive study and two to excessive fright. These cases could in no sense be considered secondary. I had in 1879 a case that was unusually free from complications and that fully satisfied me of the possibility of pelvic cellulitis as an independent affection. An unmarried lady went with her brother to an entertainment about the last of November. The rooms were crowded and hot. Having neglected to bring an extra wrap she was seized with a rigor almost immediately after going into the cold wind. Upon reaching home she retired immediately, but failed to get up satisfactory reaction for at least an hour. Upon waking in the morning she was feverish and chilly by turns. Upon attempting to dress she suffered so much pain in the pelvis that she was compelled to go back to bed. Insisting that it was only an ordinary cold and would pass off, she refused to have a physician until two days later when I was called. The pulse was small and 120. The skin was hot. She complained of great pain and tenderness of the abdomen—had most distressing dysuria and could hardly get up courage to attempt the act of defecation so great was the pain that accompanied. She said her feet were either "burning up or cold as ice." An examination per vaginam found the uterus of normal size and position, but almost immovably fixed by the dense cellular tissue surrounding it. Two months from that time every trace of the cellulitis had disappeared. The uterus was fully movable and of natural size and density. In the light of such cases we are obliged to repudiate the idea that there is no such thing as idiopathic pelvic cellulitis, although there can be no doubt that a large majority of cases are secondary to inflammation beginning in the uterus, fallopian tubes or ovaries.

A careful study of the statistics at hand places the causes of this malady in the following order, viz :

The puerperal state.
Abortion.
Operations on the uterus.
Pessaries.
Operations for hemorrhoids.

The nervous exhaustion that follows a prolonged labor coupled with the increased vascularity of the pelvic organs and the rapid metamorphosis going on in the tissues at that time would naturally lead us to expect the lying-in patient to be more subject to all forms of inflammation affecting the uterus and such other tissues as bear intimate relations to it than at other times. The statistical history of pelvic cellulitis fully justifies such expectation. Courtey estimates that about two thirds of all cases met with occur in connection with either delivery or abortion. Duncan found that of forty cases studied by him twenty-five were connected with the puerperal state. The special causes in relation to the puerperal state are laceration of the cervix, laceration of the perineum, inflammation extending from the veins of the uterus, pressure during labor and blood poisoning. In the first three instances it is a simple extension of inflammation by continuity. In the fourth it starts from a slough occasioned by too long continued pressure on a given point. Blood poisoning has not been usually mentioned as a case of cellulitis. For the simple reason perhaps that the original disease, by its appalling importance overshadows the cellulitis.

I was called in the summer of 1880 to see a lady that was said by her medical attendents to have typhoid pneumonia. To the casual observer the hacking cough,the constant fever, the dry tremulous tongue, the extreme exhaustion, the dullness on percussion over a portion of one lung certainly looked like such might be the case. But there was a suspicious odor about the bed that led me to enquire about the discharges from the uterus which elicited the information that near three weeks before she had expelled from the uterus what she termed a false conception. It turned out that she had thrown off a fœtus of a few weeks and that the placenta was still retained within the uterus in a partially decomposed state, producing a most sickening discharge. The neck of the uterus and the walls of the vagina centained several small abscesses. The placenta was extracted and the uterus washed out with a one per cent. solution of carbolic acid. The abscesses were opened and the patient placed on a supporting treatment. For three days she seemed to do well. On the morning of the fourth she complained of dysuria. A digital examination found the roof of the vagina boggy and swollen, the uterus nearly immovable. Two days later the uterus was firmly fixed and the whole roof of the vagina quite hard. As there was no inflammatory action followed the opening of the abscesses or the extraction of the placenta for nearly four days it is fair to suppose that the operations of themselves had nothing to do with the attack of cellulitis. The most reasonable supposition would seem to be that septic matter had been carried from some other abscess through the circulation or that the uterus had not been thoroughly disinfected and the matter derived from that source and deposited in the pelvic areolar tissue forming a new abscess there as the centre from which the inflammation had spread.

I was asked by a medical friend to see a lady aged about thirty for whom he had ligated two small hemorrhoidal tumors a week before. The patient complained of pain in the back and tenderness over the abdomen. She had fever and suffered from nausea. The tumors had sloughed off when I saw her. The uterus was immovable and nearly encircled by dense tender tissue. There was evident cellulitis and peritonitis present.

The diagnosis of fully developed cellulitis is not gener ally difficult. The history of the case coming on with a more or less marked rigor followed by an elevation of temperature with a small, frequent pulse associated with pain and tenderness in the lower part of the abdomen directs the attention to the pelvic organs. An examination per vaginam finds excessive tenderness at one or more points. There is a boggy feeling at first or if the case has sufficiently developed the seat of the inflammation will be easily mapped out with the finger as a mass as hard as a fibroid. At first it may be movable but if it is extensive in a few days it becomes fixed by the crowding of the over-congested surrounding tissues, or from adhesions resulting from the accompanying local peritonitis or from both these causes combined. If the inflammation involves the broad ligaments—and that is the most frequent location—the roof of the vagina will be felt as an immovable overlying stratum of dense tissue. The mobility of the uterus is almost constantly interfered with. In the majority of cases becoming almost immovably fixed after a few days.

If the diagnosis of these typical acute cases is easy that of what we may call chronic or subacute cases is often equally difficult. This is especially true of cases seen late—after they have commenced to decline. Some of them seem to creep on so insidiously that the patient can not refer you to any definite beginning. The patient will tell you that she has had no rigor or fever, but that she noticed a fortnight or more ago that there was a dragging sensation in the pelvis accompanied by backache and pain in the hips and lower limbs—that it hurt her to stand on her feet or sew on a machine, that her appetite left her and that she has "gone all to pieces" —that is her menstrual period has come on out of time and too free. She will likely speak of more or less pain and difficulty in emptying the bladder and bowels. Or she may tell you that several months since she had typhoid fever and that she has never been able to get about since on account of the pain in the hips and back. A few well directed questions will bring out the history of a clear case of acute cellulitis at the beginning mistaken by her doctor for typhoid fever. Inexcusable as this may seem I have several times known it to happen and have at this moment such a case under treatment, who five months since, was treated by an irregular practitioner for typhoid fever, when the history as related by herself and friends clearly shows that she had cellulitis from which she has not yet recovered. These cases you frequently find dragging around attending to household affairs. You introduce the finger into the vagina and swinging it around you soon find a hard mass that is more or less movable perhaps. By conjoined manipulation you make out that it is as large as a duck's egg or possibly larger—it seems to be intimately connected with the uterus; there is very little tenderness. Now how are we to know that this is not a fibroid but a case of cellulitis? If you examine the pulse of such a patient you find it small, weak and abnormally frequent, often reaching 110 per minute or more. The temperature, in spite of her statement to the contrary, will be found above the normal. If the tumor be a fibroid, uncomplicated with cellulitis, peritonitis or other acute trouble, the pulse and temperature will be normal. The following case is interesting in this connection:

February 22d, Mrs ———, æt 22, three months married. She had about a month after her marriage a considerable uterine hemorrhage that lasted for several days and was increased by being on her feet. Several times she attempted to get up, but would in a day or two have to go back to bed on account of the flow. She also had some pain in the hips and through the pelvis. An irregular practitioner was called in who said she was pregnant and about to miscarry. I found the uterus anteverted and three inches deep. The organ from fundus to os was as hard as a rubber ball. Occupying the space to the left of the uterus could be felt a hard mass that by conjoined manipulation could be traced back to a point even with the crest of the ilium. The walls of the abdomen were very thin so that the irregular outline of the tumor could be distinctly mapped out by touch and even seen when pushed up by the finger in the vagina. The woman was reduced to a mere skeleton. There was not very marked tenderness on pressure. After a careful examination I was puzzled to know what to make of it. Was it the product of peritoneo-cellulitis, a fibrous tumor or a mullitocular ovarian cyst with a very short pedicle? Notwithstanding the assertion of the mother and the patient that there had been little or no fever, I found the pulse 116 per minute and the temperature 100.6°. This fact, coupled with the additional circumstances that the tumor was apparently merged into the broad ligament at its lower extremity and but slightly movable, satisfied me that the lower portion must be the interstitial deposit resulting from inflammation and that the upper portion was likely the left ovary, fallopian tube and possibly a portion of omentum glued together by a local peritonitis. This I hoped constituted the whole trouble although I was embarassed with the thought that I could only know by waiting that there was not a fibroid or small ovarian tumor that helped to make up the mass. Rest, counter irritation with iodine tincture alternated with hot poultices, prolonged hot water injections with a carefully regulated diet, pepsin, pancreatine and maltine to assist her very feeble digestive powers were the means instituted for her cure with the most happy results. April 11th, the tumor had entirely disappeared except a portion within the broad ligament that was no longer tender. The uterus had shrunk to its normal size and regained its mobility. June 12th, I examined the patient and found no trace of the attack. This case very well illustrates the points of similarity between a fibroid and cellulitis. In both the uterus is likely to be enlarged and the menstrual flow may be too free. With a fibroid the uterus is generally movable, in cellulitis it is not.

The well known fact that the pulse rate sometimes remains above the normal after the tenderness has disappeared, is one that should not be lost sight of in obscure cases. I would suggest, as a possible explanation of this fact, that the nerves encroached upon within the area of the congested tissues, after awhile loses, to some extent, their sensitiveness from compression. It has occurred to me that the reason why we have an abscess at one time and at others escape may be from the suspension of the function of the nerves (by compression) that supply the part in this way, rendering the circulation too sluggish to maintain the vitality of the parts. Without a proper nerve supply the capilaries cannot maintain a normal circulation.

The uterus is frequently drawn out of position by the contraction of the tissues at the seat of inflammation as the disease disappears and there will sometimes remain permanent deposits of lymph. The prudent gynecologist will not fail to search for these evidences of previous cellulitis before venturing upon any irritating local treatment. He very well knows that their presence means not only that what has been may be again, but that now, even upon what would otherwise seem a very slight interference, what has been *will almost certainly be again.*

Authorities differ widely as to the relative frequency of abscess. Simpson believed that half of all cases of cellulitis terminated in suppuration. Duncan thinks suppuration much more frequent. West reports forty-three cases, in which twenty-three terminated in abscess. McClintock had thirty-seven abscesses in seventy cases. Schroeder says that in ninety two cases he has seen suppuration in but one. Baker says it is very rare except when associated with puerperal fever or pyemia. Playfair quotes these last two gentlemen, and says that abscess certainly occurs in a much larger per cent. in England than would be inferred from their account of the disease. Statistics on this point in American practice are meagre and unsatisfactory, but I believe it safe to say that in private practice in the country suppuration does not take place in more than one case in ten. The most frequent seat of abscess is in the broad ligaments, but may occur at any point liable to be affected by inflammation of this character. The pus, if left to its own course, may be discharged through the vagina, the rectum, the bladder, or burrow into the sheath of the psoas muscle, and out at the groin, or even through the walls of the abdomen. Of this last variety I had a most interesting example in 1875. The woman had a large tumor of the abdomen, and had, from her account, been subject to attacks of cellutitis for two years or longer. When seen she was very much emaciated from the drain of an abscess that had opened spontaneously in the *linea alba* about half way between the umbilicus and pubic symphysis. By introducing the finger into the vagina a hard margin with a fluctuating centre could be felt in the left broad ligament. Pressure made by the fingers on this point would force the pus out at the aperture, and occasionally a tuft of hair and small plates of bone. On the 7th of April, with the assistance of Dr. A. B. Calhoun and Dr. F. I. Welch, I made an incision from the umbilicus to within an inch of the pubic symphysis. When the fingers were introduced and a careful examination made, it was found that the tumor was a multilocular dermoid cyst the size of a man's head, springing from the left ovary. During an attack of cellulitis an abscess had formed in the left broad ligament and burst through into the abdomen near its attachment in front. Before rupturing, the accompanying peritonitis had glued fast to the abdominal wall a portion of the tumor, and hence the pus was received into this sack, from which it finally made its escape by passing directly through the abdominal wall as above stated. The tumor, after being carefully dissected off from the abdominal wall and from two points of adhesion to the omentum, was removed. The pedicle was pocketed, the abdomen carefully cleared of blood and pus, and the wound closed with silver wire. Her recovery was complete.

The after effects of cellulitis are so clearly stated by Emmett that I may be pardoned for giving it entire: "When reaction occurs, if the circumstances are favorable, the œdema of the tissues rapidly disappears, and these hard masses melt away, as it were. The uterus soon becomes again movable, and the only product of the inflammation remaining afterwards will be a band formed from the shrinkage of the tissues which had been involved. Should the uterus or intestines be bound down by adhesions the former can be relieved and replaced by art, while the peristaltic action will in time liberate the latter. But the damage will be almost irreparable if the ovaries have been involved, or the broad ligaments, if of sufficient extent to include the fallopian tubes. As the ovaries are stationary they will remain buried in the lymph that has been thrown out, and may become atrophied. * * * * Nerve filaments are often involved in the mass, and are compressed by contraction, with the effect of causing ovarian neuralgia or reflex irritation elsewhere. To attacks of cellulitis which may have produced but little disturbance at the time, can be traced the chief causes of sterility. The ovary may become covered in by a mass of lymph so that the ova cannot escape from the Graafian follicles. The fimbriated extremity of the fallopian tube may have been bound down or so displaced by adhesions as to be no longer able to grasp the ovary for the purpose of receiving the ovum as it escapes from the ovarian stroma. Or some portion of the fallopian tube may become obliterated by a band of adhesion. Moreover, these consequences are by no means dependent upon the apparent gravity of the attack.

*Treatment.*—When we look back over the history of so-called child-bed fever—many of which were doubtless cases of cellulitis, sometimes, perhaps, associated with blood-poisoning—from the day of Gordon, 1790, to 1860, and read of the heroic blood-letting, blistering, purging and profuse diuresis and diaphoresis, we can but wonder that any of the cases recovered, and need feel no surprise that the way was opened up for Homœopathy or anything else that left such cases to nature and reasonably fair hygienic surroundings.

In managing cellulitis we have but to remember that it is a simple inflammation, to be treated upon the same principles as inflammation elsewhere. The first consideration is rest in the recumbent posture—not only rest from exertion, but, as far as possible, physiological rest; and of equal importance is the early interruption of nerve conductility. These ends will both be best attained by the administration of full doses of morphia, which has the additional advantage of relieving the pain. Emmett lays great stress on the abortion of cellulitis when seen early, by the use of hot water thrown into the vagina for several hours at a time. I have never given it a fair trial, but can testify to the beneficial effect of hot water injections of half an hour's duration, which I have seen used with good results at every stage of the affection. When hot water is used it should always be borne in mind that it must be administered with the least possible disturbance to the patient. It is better not to attempt its use if it must be left to a clumsy, inexperienced nurse. Large hot poultices over the abdomen do good unquestionably. Whether they act by simply diverting a certain amount of the circulation from the inflamed tissues within, or owe a portion of their results to the effects of heat upon the contiguous nerves and nerve centres, I know not, but of the clinical fact that they do good I feel quite certain. They should be large and as hot as can be borne, and should be renewed every hour. After the active inflammation has subsided painting the abdomen with iodine tincture seems to be of some benefit.

Should an abscess form the same rules apply to it here as elsewhere. Dr. Brickell, of New Orleans, a few years since, advanced the idea that there are two distinct forms of pelvic inflammation, serous and purulent. This view has since been sanctioned by some of our leading authorities, and has not been denied, so far as I know, by any. The presence of fluid, whether serous or purulent, when detected should be promptly dealt with, either by incision or aspiration. If the abscess be so situated as to admit of a free incision, that is the better plan to follow. If purulent, the cavity should be washed out daily with an antiseptic solution. In severe cases, and especially if accompanied with purulent infection, it will severely test the vital powers of the patient. Of course, such cases must receive a carefully directed supporting treatment. Brandy, with cod liver oil, will be found especially serviceable to those whose stomachs will bear it. Iron is not well borne until convalescence is fairly established. Quinine is of signal benefit throughout the disease. Administered in small doses two or three times daily, it assists the digestion, keeps up the tone of the nervous system and lowers the temperature when it acts on the skin.

